# Donor–acceptor complexes between photoinitiators and hybrid organic–inorganic SZ2080™ photoresist

**DOI:** 10.1039/d5ma01526j

**Published:** 2026-03-13

**Authors:** Marius Navickas, Dimitra Ladika, Edvinas Orentas, Martynas Talaikis, Gediminas Niaura, Mantas Grigalavičius, Mantas Gaidys, Ricardo J. Fernández-Terán, Mangirdas Malinauskas, Mikas Vengris

**Affiliations:** a Vilnius University, Laser Research Center Saulėtekio av. 10 LT-10223 Vilnius Lithuania marius.navickas@ff.vu.lt; b Center for Physical Sciences and Technology Saulėtekio av. 3 LT-10257 Vilnius Lithuania; c Department of Physical Chemistry, University of Geneva. Quai Ernest-Ansermet 30 CH-1205 Geneva Switzerland Ricardo.FernandezTeran@unige.ch

## Abstract

Multi-photon photopolymerisation (MPP) based on organic–inorganic resins has emerged as a promising technique for the fabrication of complex three-dimensional nano- and micro-structures. Among the prepolymers used for MPP, SZ2080™, synthesised *via* sol–gel method, stands out for its hybrid nature, comprised of an organic and inorganic network. This photoresist is usually photosensitised with the photoinitiators IRG369 and Michler's ketone, that are responsible for initiating polymerisation. The incorporation of these photoinitiators induces significant changes in the ground-state absorption of the material and enables a broad fabrication window. Despite its broad application, the fundamental processes governing the performance of SZ2080™ remain poorly understood. In this study, we systematically investigate the optical characteristics of SZ2080™ sensitised with IRG369 and Michler's ketone using UV/Vis absorption, steady-state fluorescence, Raman, along with Fourier-transformed infrared and nuclear magnetic resonance (NMR) spectroscopies. The UV/Vis spectra reveal pronounced alterations in ground-state absorption, suggesting coordination-related interactions. Vibrational spectroscopy further supports these findings, indicating that the observed modifications are linked to changes in the inorganic network of SZ2080™. Finally, ^1^H NMR confirms that the diethylamino groups of Michler's ketone, along with the carbonyl groups of IRG369, coordinate with the Zr(iv) centres in the SZ2080™ matrix. The link between spectroscopic characterisation and MPP fabrication highlights that controlled complex formation could greatly improve the fabrication window.

## Abstract 2

Michler's ketone is one representative photointiator able to form a donor–acceptor complex with SZ2080™ prepolymer, thereby enhancing the photopolymerisation performance.

## Graphical abstract

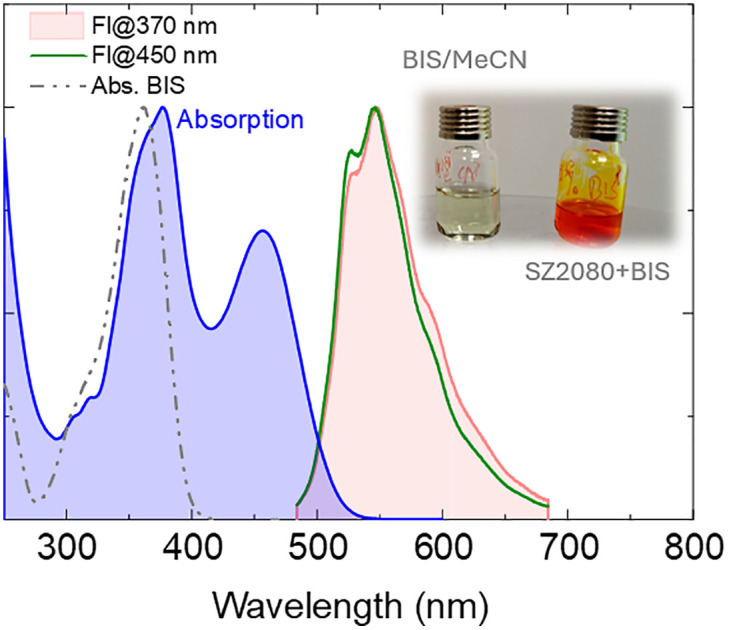

## 1 Introduction

Additive manufacturing *via* multi-photon polymerisation (MPP) became a powerful microfabrication technique, where tightly focused femtosecond laser pulses initiate localised polymerisation within photosensitive materials, allowing for precise, direct-write fabrication of high-resolution three-dimensional (3D) architectures.^1–6^ Historically, the first class of materials used for MPP, was acrylates.^7^ This class of material possesses high photoactivity and chemical tunability.^8,9^

In pursuit of the synthesis of materials for MPP lithography with desired properties, a negative photoresist, SU-8, was developed in 1996. SU-8,^10^ designed for precise UV-aided photostructuring, was naturally adopted for MPP lithography applications. It is a chemically amplified photoresist that, upon exposure to light, generates a small amount of a strong acid, a precursor to the cross-linking process. For this reason, synthesis of SU-8 relies on acid-labile groups and a photoacid precursor, leading to a rather strict processing protocol: annealing to remove solvent and UV exposure to generate acid (like hexafluoroantimonic acid), and cross-linking of epoxides through protonated oxonium ions.^11^

In parallel, hydrogels were developed as a class of photosensitive materials for MPP, especially attractive for biomedical applications.^12^ Hydrogels are polymeric networks that can absorb and hold large amounts of water within their 3D structures, due to their hydrophilic functional groups.^13,14^ They can be derived from natural or synthetic sources and tailored in composition (homopolymeric, copolymeric, interpenetrating networks), crystallinity, or ionic properties. In MPP lithography, a common photopolymerisable hydrogel is PEGDA,^15^ which consists of polyethylene glycol with two acrylate substituents at the ends of the chain.

Although acrylates involving hydrogels as well as SU-8 have defined much of the foundational work in MPP, more recently, a class of organically modified silicates (ORMOSELs) has been developed. Among these, SZ2080™—a zirconium–silicon-based hybrid prepared *via* sol–gel has emerged as one of the most promising materials for advanced photopolymerisation.^16,17^ The SZ2080™ was fundamentally designed for a 780 nm MPP wavelength; thus, this material usually works with Ti:sapphire lasers. However, recent studies^18–21^ have shown that other wavelengths, such as 1030 nm and its second harmonic at 515 nm, obtained from commercial Yb:KGW lasers, are also suitable and trigger the photopolymerisation reaction. This discovery made SZ2080™ a versatile material for additive manufacturing with near-IR laser sources.

SZ2080™ also provides access for tunability of the refractive index by changing the ratio between the organic–inorganic parts.^17^ Combining the structural rigidity and chemical stability of inorganic networks with the cross-linking flexibility of organic groups, SZ2080™, features the ability to downscale the structures,^17^ followed by relatively high optical transparency in the wavelength regime where MPP usually operates (780 nm).^22^ Versatility of SZ2080™ has made it a material of choice across diverse applications, including photonics,^23^ micro-optics,^24^ micromechanics,^25^ and bio-applications.^26^

SZ2080™ sensitised with common photoinitiators IRG369 [2-benzyl-2-(dimethylamino)-1-(4-morpholinophenyl)butan-1-one] and BIS [4,4′-bis(diethylamino)benzophenone, Michler's ketone]^1^ shows exceptionally rapid and efficient photopolymerisation.^27^ Additionally, these photosensitive mixtures show unusual changes of UV/Vis absorption and emission.^28^ Although these changes have been observed and documented, no clear link between spectral changes and polymerisation parameters has been established so far. Understanding this phenomenon is crucial, as it could lead to new potential functionalities, improved process maintenance, and expanded capabilities for hybrid resin-based systems in advanced MPP. Therefore, in this work, we present a systematic study of SZ2080™ with the incorporation of IRG369 and BIS photoinitiators to elucidate their coordination behaviour in the organic–inorganic prepolymer.

## 2 Experimental details

### 2.1 Materials

The hybrid organic–inorganic prepolymer SZ2080™ was prepared as discussed in ref. 29, and mixed with 3 wt% of photoinitiators (PIs) 2-benzyl-2-dimethylamino-1-(4-morpholinophenyl)-butanone-1 (IRG369) and 4,4′-bis(diethylamino)benzophenone (BIS). In general, SZ2080™ is synthesised from methacryloxypropyltrimethoxysilane (MAPTMS), methacrylic acid (MAA), and zirconium *n*-propoxide (ZPO). The photoinitiators (PIs) IRG369 and BIS were purchased from Sigma Aldrich and used without further purification.

Two types of samples were prepared for UV/Vis characterisation: one of thin films on a thick UV-fused silica glass substrate, and the other solutions of HPLC-grade acetonitrile (MeCN, Sigma Aldrich), each with a different PI. The solutions were further diluted to an optical density less than 1 OD in a 2 mm quartz cell (Hellma). Thin films were fabricated on fused-silica glass substrates to measure ground-state absorption and steady-state fluorescence of photosensitised SZ2080™. The photosensitised mixture was prepared by adding photoinitiator (PI) into SZ2080™ and stirring with a magnetic stirrer until the PI dissolved. Films made of SZ2080™, mixed with 3 wt% of IRG369 and BIS, were prepared by spin coating. Stimulated Raman experiments were conducted for sensitised SZ2080™, measuring their spectra in 2 mm quartz cuvettes (Hellma) while neat PI Raman spectra were measured for acetonitrile 3 wt% solutions. Because the Raman signals were very weak, a higher PI concentration (3 wt%) was required. Although the concentration used in these experiments is slightly higher than that typically used in MPP fabrication, they still remain reasonably close to practical values (1 wt%). All photoinitiators are fully soluble in SZ2080™ at the concentrations used (1–3 wt% relative to the monomer fraction), yielding optically transparent and stable formulations.

### 2.2 MPP fabrication

To establish the baseline for different performance of two types of PIs, two pairs of widely used materials were used in MPP experiments. BIS and IRG369 were chosen as representatives of ‘complex forming’ PIs, whereas TPO and BAPO represented ‘simply mixing’ PIs. The separation was based on whether the Raman spectra of prepolymer/PI mixtures were significantly different from the difference spectra of PI and prepolymer (see supplementary material). The samples for laser structuring were prepared by drop-casting the synthesised SZ2080™ onto thin glass substrates. The deposited droplets were then placed under vacuum at room temperature for 24 hours to promote densification by solvent removal. This densification step minimises shrinkage during laser exposure and subsequent development. The densified droplets were then used to fabricate resolution bridges, followed by development in 4-methyl-2-pentanone for 30 minutes under ambient laboratory conditions. The resolution bridge lines (15 µm) were fabricated using a galvo-scanner at a scanning speed of 100 µm s^−1^, when focussing with a 63×/1.4 NA objective lens (Plan- Apochromat, Zeiss) and using Immersol™ 518F immersion oil. The laser power was varied from 0.2–2.2 mW, corresponding to an average pulse intensity of *I* = 0.37–4.02 TW cm^−2^ with a constant 200 kHz pulse repetition rate and a pulse duration of 300 fs. The intensity calculations are listed in the SI. Finally, the fabricated samples were coated with 10 nm Ag and characterised using a scanning electron microscope (SEM, JEOL JSM-6390LV). By varying the laser power, the dynamic fabrication window (DFW)^27^ was estimated for each photoinitiator.

### 2.3 Spectroscopy

Ground-state absorption spectra of thin-films and MeCN solutions were recorded using a Shimadzu UV-3101PC UV/Vis absorption spectrometer. Steady-state emission and emission excitation spectra of both types of samples were measured using a Cary Eclipse emission spectrophotometer. The emission spectra of PI solutions were measured on *ca.* 0.2 OD samples at right angle with respect to the excitation light, using standard 1 cm optical pathlength quartz cuvettes (Hellma). The emission spectra of SZ2080™ sensitised with PIs were measured on *ca.* 0.3 OD spin-coated films, at the same excitation/detection configuration as used for PI solutions. Suitable filters were placed in the emission monochromator to collect excitation/emission spectra without artifacts.

Precise UV/Vis titration experiments were carried out to corroborate the formation of the 1 : 1 donor–acceptor complex. The titration experiment was performed for both IRG369 and BIS stock solutions by using the 0.377 mM initial PI concentration and 0.0225 M concentration of Zr(iv) in SZ2080™. The solution was prepared by adding the appropriate amount of the SZ2080™ aliquot to acetonitrile (MeCN) to account for up to 40 equivalents of Zr(iv) atoms during 20 titration repetitions. We also ensured that the concentration of PI remained unchanged compared to the analyte solution.

Ground-state stimulated Raman scattering (SRS) spectra of each compound were measured using a Light Conversion Harpia-TA commercial spectrometer designed for three-pulse transient absorption, including femtosecond stimulated Raman scattering (FSRS) measurements. In the SRS experiment, we used only two pulses of parallel polarisations—Raman pump and broadband probe—to capture the ground-state vibrational modes. When the broadband femtosecond continuum pulse is overlapped in the sample with the narrowband (picosecond) Raman pump, the vibrational resonances of the sample amplify the continuum spectrum at the frequencies corresponding to the differences between the Raman pump and vibrational modes of the sample. Typically, the duration and spectral with of the Raman pump is 2.0 ps and 10–20 cm^−1^, respectively. In our SRS experiments, the Raman pump pulse was generated by spectral filtering of the fundamental Ti:Sapphire (Libra, Coherent, Ltd) radiation through a Fourier-4f filter. The 1.0 ps duration of the Raman pump pulse was estimated as the full width at half maximum (FWHM) value of the cross-correlation between the Raman pump and probe pulses.

A broadband Raman probe was produced in a 5 mm YAG crystal, to obtain an efficient spectral broadening in the infrared region. Since the Raman pump was at 800 nm, all Stokes SRS components were shifted to the red, relative to 800 nm. The probe light was further spectrally filtered from the excessive 800 nm radiation, using a long-pass glass filter (Shott RG830, *T*_830–2700nm_ = 85%). After passing through the sample (2 mm Hellma quartz cuvette), the probe beam is re-collimated and focused into the entrance slit of an imaging spectrograph, where it is read out with a 256-pixel photodiode array. To enhance the spectral resolution (up to 3.5 cm^−1^), a 1200 mm^−1^ diffraction grating (Thorlabs GR25-1210) was used to disperse the Raman probe spectrum. The wavenumber axis of the spectra was calibrated using toluene as a reference. The other details regarding the operation of SRS set-up can be found in ref. 30. The datasets associated with 830 nm spontaneous Raman excitation were collected to acquire a broader range compared to SRS using an inVia Raman spectrometer (Renishaw, UK) equipped with a confocal microscope (Leica, Germany) and a thermoelectrically cooled CCD detector operating at −70 °C. Spectra were obtained with 16 mW excitation power using a 50× and 0.5 NA objective (dedicated for IR) and an integration time of 10 minutes. The wavenumber axis of the spectra was calibrated according to the silicon 520.7 cm^−1^ band.

Fourier-transform infrared (FTIR) spectra were acquired in transmission mode using an Alpha spectrometer (Bruker, Germany). Samples consisting of 10 µL of PI mixed with either acetonitrile or SZ2080™, were placed between two CaF_2_ windows. Spectra were recorded using 20 scans at a resolution of 4 cm^−1^.

Nuclear magnetic resonance (NMR) spectra were recorded on Bruker DRX400 instruments in CDCl_3_ and CD_3_CN and were calibrated using residual undeuterated solvent as an internal reference (CDCl_3_: ^1^H NMR *δ* = 7.26 ppm, ^13^C NMR *δ* = 77.16 ppm). DOSY (see SI) experiments were performed on a 400 MHz Bruker Avance NMR spectrometer equipped with an Accustar *z*-axis gradient amplifier and an ATMA BBO probe with a *z*-axis gradient coil. All experiments were run using insert tubes and without spinning to avoid convection. All calculations were performed using standard applications in Bruker Topspin and MestReNova software. Diffusion was measured at 22 °C using standard Bruker pulse sequence (stegp1s), employing a stimulated echo sequence and 1 spoil gradient with a diffusion gradient, *δ*, set to 2 ms and the diffusion time, *Δ*, to 120 ms. The rectangular gradient pulses applied ranged from 2–98% of the maximum gradient output of 48.15 Gauss per cm, in 32 gradient steps. Individual rows of the quasi-2D diffusion databases were phased, baseline-corrected and aligned. At least three peaks were analysed for each compound. NMR titrations were performed by adding aliquots of neat SZ2080™ into a CD_3_CN solution (24 mM) of BIS or IRG369.

### 2.4 Density functional theory calculations

To predict the Raman spectrum of SZ2080™ and ascribe the bands to physical vibrations, quantum-chemical calculations were performed with Gaussian 09 D.01.^31^ Molecular geometries were optimised without symmetry constraints using density functional theory (DFT) with the B3LYP exchange–correlation functional and the 3-21G, def2-TZVPP basis sets. Vibrational frequencies and Raman activities were computed at the same level of theory. Simulated Raman spectra were produced by converting Raman activities to intensities, applying a Lorentzian broadening with full width at half maximum (FWHM) of 10 cm^−1^.

## 3 Results and discussion

### 3.1 MPP fabrication

**Fig. 1:**
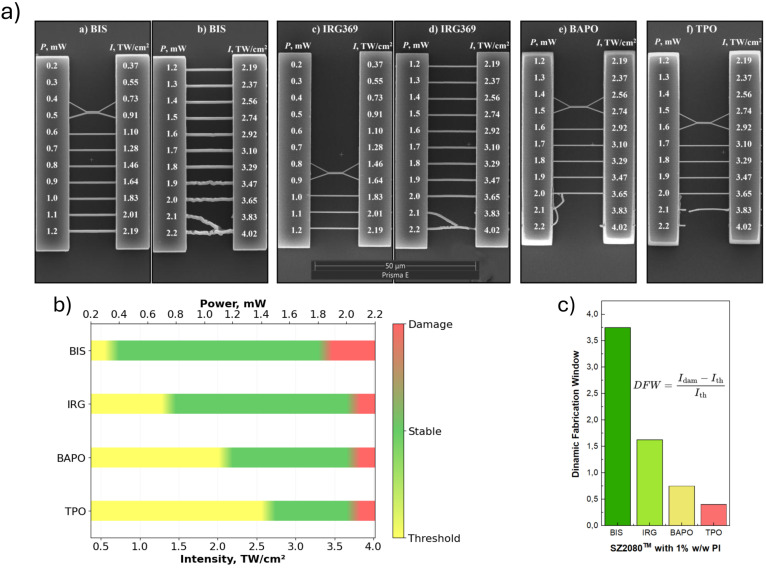
(a) Scanning electron microscope images of resolution bridges on SZ2080™ sensitised with IRG369, BIS, BAPO and TPO PIs, (b) laser processing window for SZ2080™ with different PIs and (c) dynamic fabrication window values for SZ2080™ with different PIs.

The resolution bridges were fabricated to assess the laser fabrication window as well as the dynamic fabrication window (DFW) of sensitised SZ2080™ with IRG369 and BIS PIs. The results are presented in Fig. 1. For comparison, we used BAPO and TPO as reference PIs that do not form any complexes with SZ2080™. The dependence of PI on the processing of the SZ2080™ with 1030 nm laser irradiation is shown Fig. 1B and C. At first glance, the structures fabricated with BIS and IRG369 sensitised prepolymer are available in a broader region of laser radiation intensities, than for BAPO and TPO. This is visualised by the processing window, presented in Fig. 1B. The particular data indicates that BIS allows to fabricate 3D structures even at lower *I*_th_ – 0.55 TW cm^−2^ intensities, while for IRG369, *I*_th_ is equal to 1.46 TW cm^−2^. Both BAPO and TPO need quite a bit higher intensities for line fabrication with threshold intensities equaling to 2.19 and 2.74 TW cm^−2^, respectively. Comparing the broadness of DFWs (Fig. 1B), BIS and IRG369 possess the highest values of 3.5 and 1.63, respectively, compared to BAPO and TPO, which feature only 0.75 and 0.4, respectively. An important point here is that the structures, fabricated in the ‘burning regime’, are still usable upon fabrication with BIS and IRG369 PIs. In contrast, the lines fabricated under the same conditions with BAPO and TPO have burnt and were washed out during the development step.

### 3.2 UV/Vis absorption spectroscopy

**Fig. 2:**
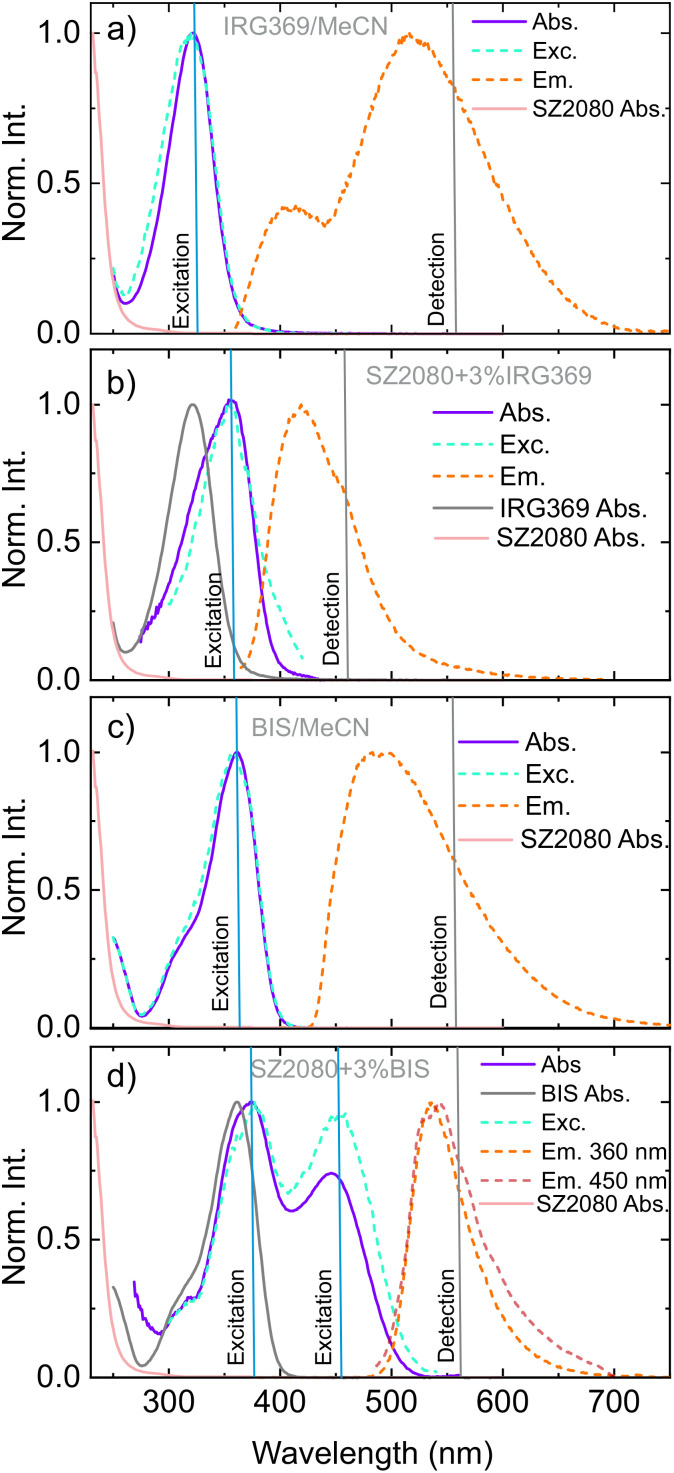
Ground-state absorption and steady-state emission spectra of (a) IRG369 and (c) BIS compounds, dissolved in acetonitrile. Panels (b) and (d) represent the change of the ground-state absorption and fluorescence spectra when PIs are embedded to SZ2080™. The grey lines represent the comparison between the UV/Vis spectra of the PI and the photosensitised SZ2080™, while the cyan lines in all panels correspond to the fluorescence excitation profiles. Vertical lines show the wavelengths used to excite or detect the corresponding emission/excitation spectra.

MPP indicates an unusual behaviour of IRG369 and BIS PIs that enhance the photopolymerisation window. The DFW in both PIs’ cases was significantly improved compared to using BAPO and TPO. To learn about those reasons, the ground-state absorption and steady-state fluorescence spectra of the corresponding samples were measured. The absorption and emission spectra of IRG369 dissolved in acetonitrile (MeCN) are presented in Fig. 2A. The ground-state absorption of IRG369 was previously attributed to electronic transitions from occupied π states to unoccupied π* states of the aromatic C=C bonds.^28,32,33^ At first glance, the emission of IRG369 in MeCN features dual bands and is anomalously broad; it stretches over almost the entire visible range (from 360 nm to 700 nm), showing an extremely large Stokes shift. The latter feature of IRG369 ground-state absorption might be a consequence of a change in solvent (environment) polarity. The fluorescence excitation profile (Fig. 2A, cyan curve) overlaps almost perfectly with the ground-state absorption, thus confirming that the observed emission is a direct result of the molecule absorption. However, the drastic change in the emission spectra when IRG369 is embedded to prepolymer (now featuring a maximum at 420 nm) suggests that this effect is not purely the change of dielectric constant, strongly hinting at the formation of a radiative complex. Moreover, the Stokes shift of the emission in SZ2080™ mixture itself is still quite large (5860 cm^−1^), atypical for most organic molecules. This unusually large Stokes shift might be related not only to the formation of a complex, but also indicate the formation of a complex featuring a ligand to metal charge-transfer (LMCT) state, likely influenced by the Zr(iv) atom in the inorganic network of SZ2080™. The excitation spectrum (Fig. 2B) differs slightly on the red-edge of the ground-state absorption, indicating inhomogeneity in the ground state.

Addition of 3% BIS to the neat SZ2080™ prepolymer, yields a new absorption band centred around 450 nm, which is absent in the neat BIS compound dissolved in MeCN (see Fig. 2C and D). This new band likely arises from charge transfer interactions between the electron-rich Michler's ketone and Lewis acidic Zr(iv) centres of the SZ2080™ inorganic network.^28^ The fluorescence excitation spectrum differs from the lowest ground-state absorption, indicating different emission yields exciting at both bands. According to M. Stavrou *et al.*, the observed absorption and emission changes might arise from several factors:^28^ sensitivity of PI to different dielectric constant, interactions between PI and the organic methacrylate group in SZ2080™, and interactions between the amine groups of the PIs and the inorganic part of SZ2080™.

### 3.3 Vibrational spectroscopy

**Fig. 3:**
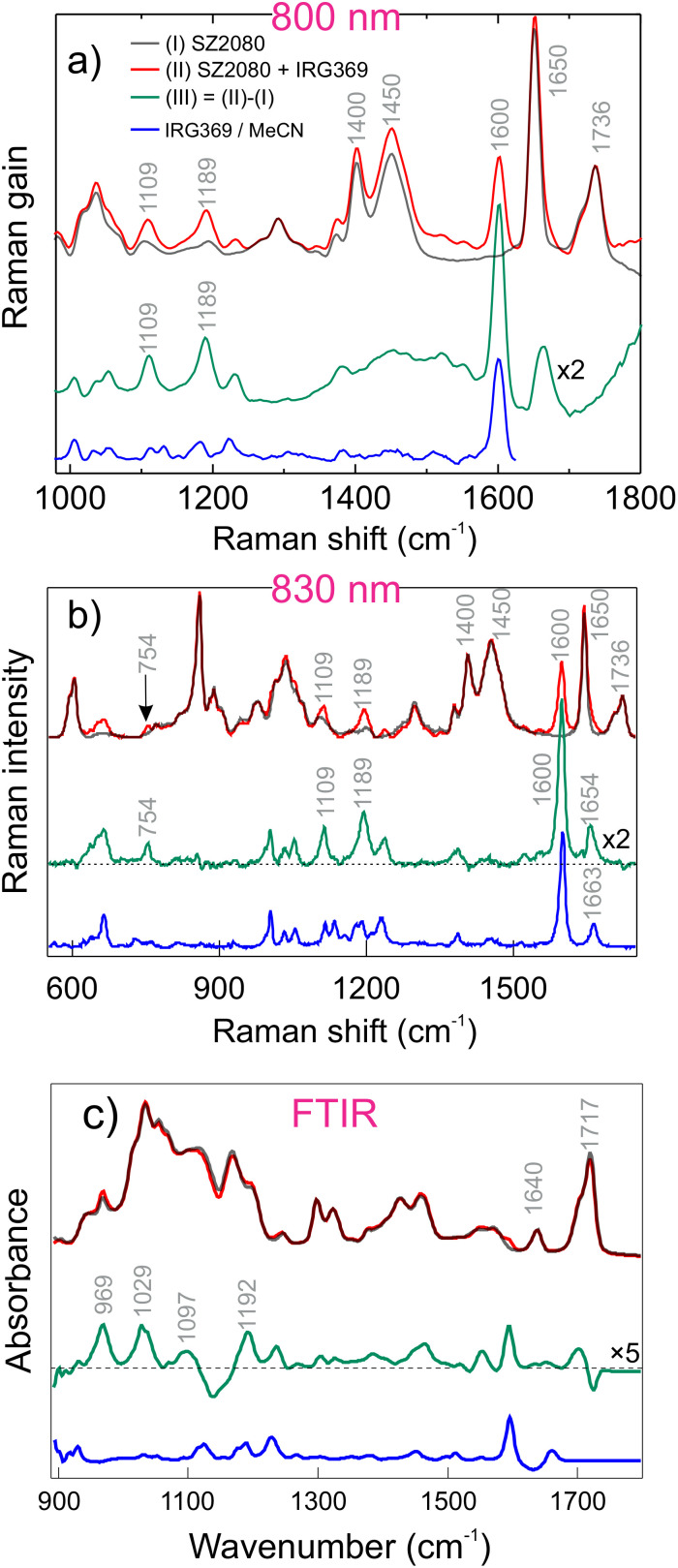
(a) SRS spectra of neat (black) and SZ2080™ sensitised with IRG369 (red). The blue curve represents the SRS spectrum of neat IRG369 dissolved in MeCN. Panel (b) shows the spontaneous Raman datasets acquired in a broader range using 830 nm excitation while the panel (c) shows the FTIR spectra of the pure, sensitised SZ2080™ and neat IRG369. The green curve corresponds to the differential spectrum between the mixture and pure SZ2080™.

In order to learn more about the structural changes after mixing the PI with SZ2080™ and make a link with UV/Vis spectra, we conducted stimulated Raman scattering (SRS), conventional Raman scattering, and FTIR measurements. The measured vibrational spectra of neat and photosensitised SZ2080™ are depicted in Fig. 3. We can first consider the features observed in SRS spectra (Fig. 3A). In both the free PI solutions and in those containing 3% of IRG369, SZ2080™ SRS spectra demonstrate two characteristic vibrational bands at 1650 and 1736 cm^−1^. The first one is attributed to C=C stretching mode of the cross-linking methacrylate group, and another to the stretching mode of the carbonyl group (C=O).^34^ In the spectrum of IRG369-sensitised SZ2080™ (red curve), there is an additional band at 1600 cm^−1^ which is a fingerprint of the aromatic ring vibrations of IRG369.^34^ Of particular interest is the region comprising 960–1200 cm^−1^, which, according to our DFT calculations (see the text bellow for more details) and previous observations,^35,36^ contains the vibrations of the inorganic network, mostly linked in this case to various types of Zr–Si–O modes. More specifically, this region contains the bands around 1109 cm^−1^ and 1189 cm^−1^ that increase slightly in magnitude, more than can be expected from the Raman spectrum of pure IRG369. These changes are illustrated by the differential spectrum (green curve). The emergence of these new vibrations might indicate a product pre-existing after mixing these compounds together, or could correspond to the same IRG369 bands, now shifted by the change of dielectric environment. Moreover, the negligible shift of the C=C mode (1663 cm^−1^ → 1654 cm^−1^ in Fig. 3B) of IRG369 is not unambiguous, and might indicate some coordination interactions as well.

**Fig. 4:**
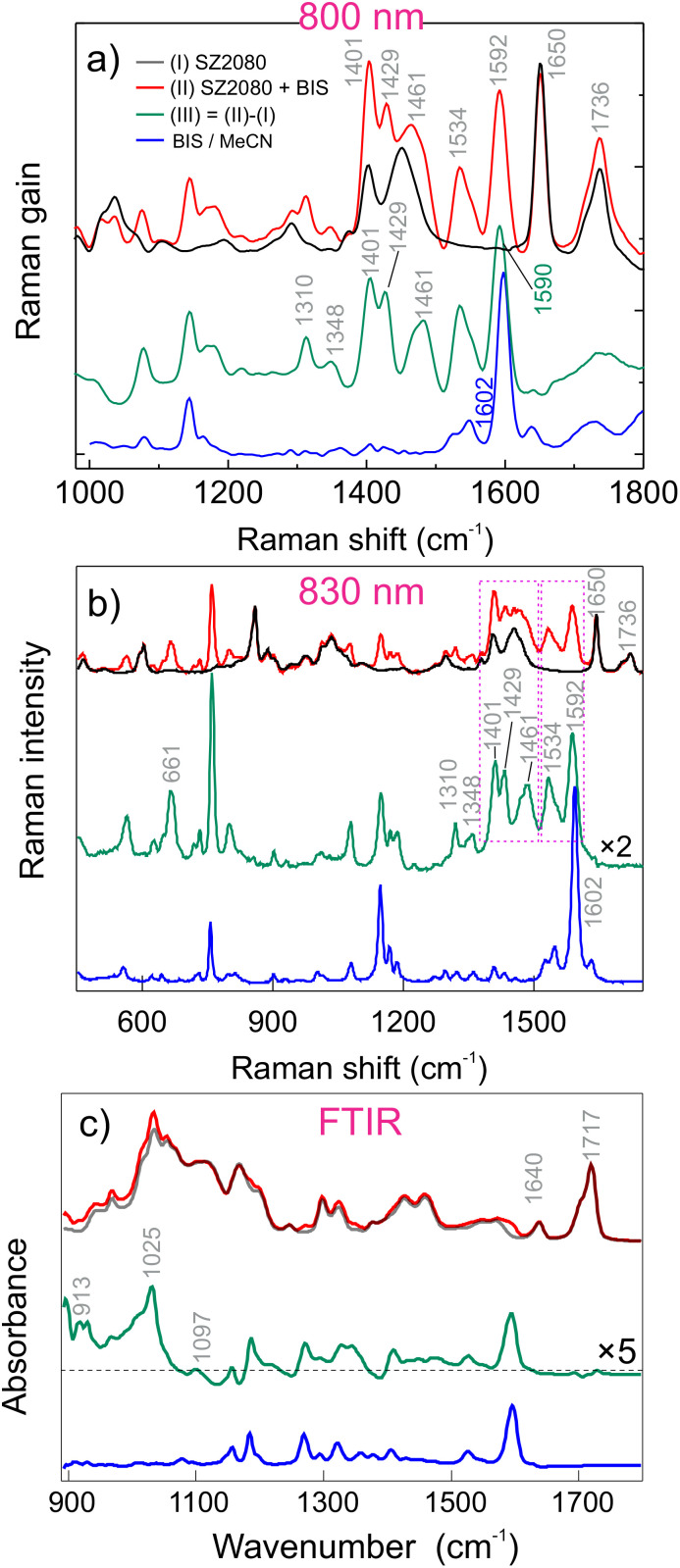
(a) SRS spectra of neat (black) and SZ2080™ sensitised with BIS (red). The blue curve represents the SRS spectrum of neat BIS dissolved in MeCN. Panel (b) shows the spontaneous Raman datasets acquired in a broader range using 830 nm excitation while the panel (c) shows the FTIR spectra of the pure, sensitised SZ2080™ and neat BIS. The green curve corresponds to the differential spectrum between the mixture and pure SZ2080™.

Conventional Raman spectra (Fig. 3B) were collected in the broader spectral region using 830 nm Raman excitation wavelength, revealing mostly the same spectral characteristics in 960–1200 cm^−1^ region; however, there is an additional vibrational band around 754 cm^−1^ in the difference spectrum that is not attributed to either of the pure components. A small shift of the IRG369 C=O stretching mode is also observed in the difference spectrum, going from 1663 cm^−1^ (see the blue spectrum) to 1654 cm^−1^ (see the green spectrum) that might be used as another peace of evidence for possible coordination. Along with the discussed observations, FTIR spectra also show similar changes upon addition of IRG369: there are specific bands at 969, 1029, 1097 and 1192 cm^−1^ (green curves in Fig. 2C). These bands are missing in the neat IRG369 FTIR spectrum or show a different lineshape than the original IRG369 (the band at 1192 cm^−1^). No changes occur on the methacrylate groups featuring FTIR bands around 1717 cm^−1^ and 1640 cm^−1^, suggesting that all potential interactions of SZ2080™ with the PIs should take place *via* the inorganic framework (see Fig. 3C and 4C).

In previous work, M. Stavrou *et al.*^28^ postulated that confinement of IRG369 in the SZ2080™ prepolymer might lead to interactions between the PI and the organic methacrylate group in SZ2080™. From our Raman data, we did not observe any signatures of interactions between the organic moiety of SZ2080™ and IRG369. This itself suggests that the organic framework does not participate actively in the complexation. In turn, we performed an additional experiment on methyl methacrylate (MMA) sensitised with IRG369 to finally check its binding affinity and make a link with the methacrylate in SZ2080™. As presented in Fig. S1, no binding feature was found in MMA + IRG369, which indeed suggests that the organic part is not responsible for complex formation.

The Raman features of BIS-sensitised SZ2080™ are three characteristic bands at 1592, 1650, and 1736 cm^−1^, corresponding to the previously discussed vibrational modes. Notably, pure SZ2080™ exhibits two bands at 1400 and 1450 cm^−1^ (the corresponding frequencies are indicated in Fig. 3A and B), whilst in the prepolymer mixture (red spectrum of Fig. 4A and B), it splits to a triplet of 1401, 1429, and 1461 cm^−1^, along with a shift of the aromatic ring band of BIS from 1602 cm^−1^ to 1592 cm^−1^ (see Fig. 4A and B). Since the aromatic ring features high electron delocalisation and is close to the diethylamino group—which can potentially bind to Zr(iv) by sharing electrons—the electrons in the ring will also be influenced by coordination. The differential spectrum highlights additional vibrational modes at 1348 and 1310 cm^−1^, which are absent in the Raman spectrum of pure BIS (blue curve). In the datasets acquired in the broader range using 830 nm excitation, we recognise a clear peak at 661 cm^−1^ that is not obtained in the neat BIS spectrum as well, again pointing to formation of ground-state complex. Like for IRG369, the FTIR difference spectrum (Fig. 4C) of BIS + SZ2080™ clearly shows a new, broad structured band peaking at 1025 cm^−1^, originally absent in the spectrum of pure BIS. Less pronounced bands at 913 cm^−1^ and 1097 cm^−1^ are also observed.

An important aspect determining the formation of complexes is solubility of PIs in SZ2080™. The absence of scattering artefacts in UV/Vis spectra (Fig. 2), together with well-defined Raman and FTIR differential features (Fig. 3 and 4), confirms homogeneous molecular dispersion rather than phase separation. Since donor–acceptor complex formation requires direct orbital overlap and electronic interaction, these spectroscopic observations indirectly validate sufficient solubility. Furthermore, the reproducible polymerisation thresholds and dynamic fabrication windows (Fig. 1) exclude solubility-limited artefacts.^28,37,38^

### 3.4 Nuclear magnetic resonance spectroscopy

The structure and chemical homogeneity of the synthesised SZ2080™ were confirmed by ^1^H NMR spectroscopy (see Fig. S2 of SI). The spectrum displayed all characteristic resonances with the expected integral values corresponding to the methacrylate and 3-(trimethoxysilyl)propyl methacrylate moieties, namely the overlapping resonances of the vinyl (1, 8) hydrogens, methyl groups (2, 7), silicon-bound methoxy groups (6), and signals from the alkyl chain (3–5). Sharp resonances attributable to the low-molecular-weight byproduct, propanol, were also clearly observed. To further corroborate the spectral assignments, DOSY NMR was performed to estimate diffusion coefficients for each set of resonances (see Fig. S3 in the SI). Diffusion coefficients of 1.39 × 10^−9^ m^2^ s^−1^ and 2.89 × 10^−10^ m^2^ s^−1^ were obtained for SZ2080™ and propanol, respectively, confirming that the propanol molecules are not bound to SZ2080™.

**Fig. 5:**
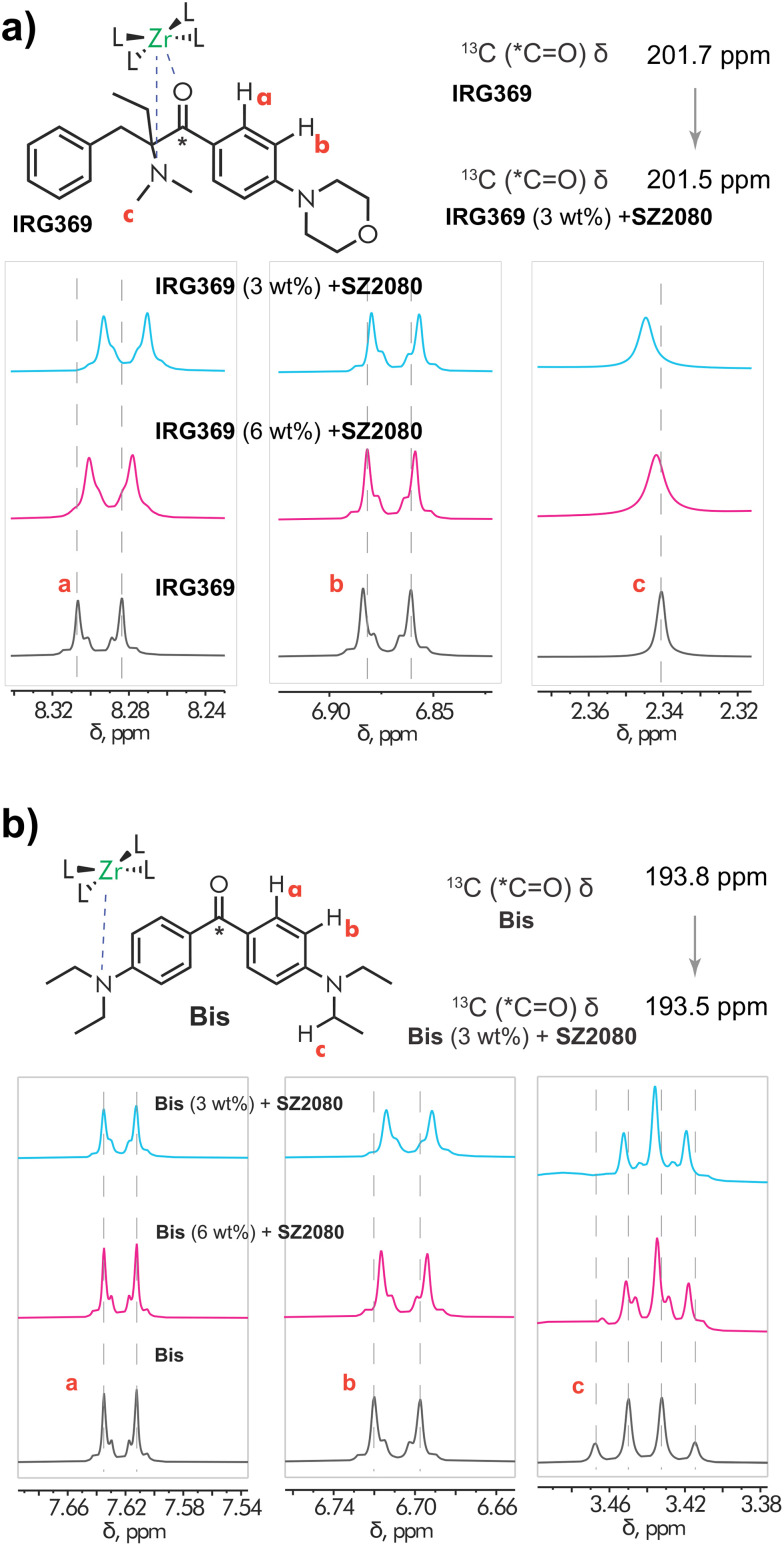
^1^H NMR (CD_3_CN) titration experiments of (a) IRG369 and (b) BIS with SZ2080™.

The structure of SZ2080™ seems to be favourable for the coordination with a suitable photosensitizer. The Zr–Si–O network, consisting of Zr(iv) (a strong Lewis acid), that accepts an electron from a donor nitrogen atom of the PIs, facilitates formation of a donor–acceptor complex in both BIS and IRG369 cases. This is not immediately clear from Raman and FTIR data, whilst UV/Vis absorption spectroscopy clearly evidences the formation of a ground-state product upon addition of PIs to SZ2080™. Here, the straightforward observation regarding the formation of donor–acceptor complexes, and even the assessment of their possible structure(s) in each case were achieved by NMR titration experiments (see Fig. 5A and B). In the case of IRG369 (Fig. 5A), an upfield shift of the aromatic ring resonances a and b were observed, with the largest change occurring for hydrogens a in proximity to the carbonyl group (Fig. 5A). The observation that the resonances of the N–Me groups were also affected suggests possible chelation of Zr(iv) by both the carbonyl and NMe_2_ groups. A small shift was also observed for the carbonyl carbon in the ^13^C NMR spectrum (Fig. S4, see Fig. S4, S6 for entire NMR spectra).

Interestingly, for the BIS photoinitiator, the NMR titration showed a shift only for hydrogens adjacent to the NEt_2_ substituent (*H*_b_), suggesting preferential binding of the amine group to the metal centre (Fig. 5B). Consistent with this, an upfield shift of the N–CH_2_–hydrogens was observed in the ^1^H NMR spectrum. The small shift observed in the ^13^C NMR spectrum is most likely a result of perturbed electron density within the push–pull carbonyl–amine system upon complexation (Fig. S4B). Another prominent feature that provides additional evidence for the formation of a donor–acceptor complex, is a change of multiplicity of the BIS N–CH_2_ protons signal (Fig. 5B). The observed spectral changes are most likely attributed to restricted rotation about the C–N bond upon complexation, involving the nitrogen atom. Collectively, these data suggest that in CD_3_CN, Zr(iv) preferentially binds to the amino group, which, in the case of IRG369, is due to the proximity of the carbonyl and amino functionalities, also potentially results in chelate formation.

### 3.5 UV/Vis titration and competitive binding

**Fig. 6:**
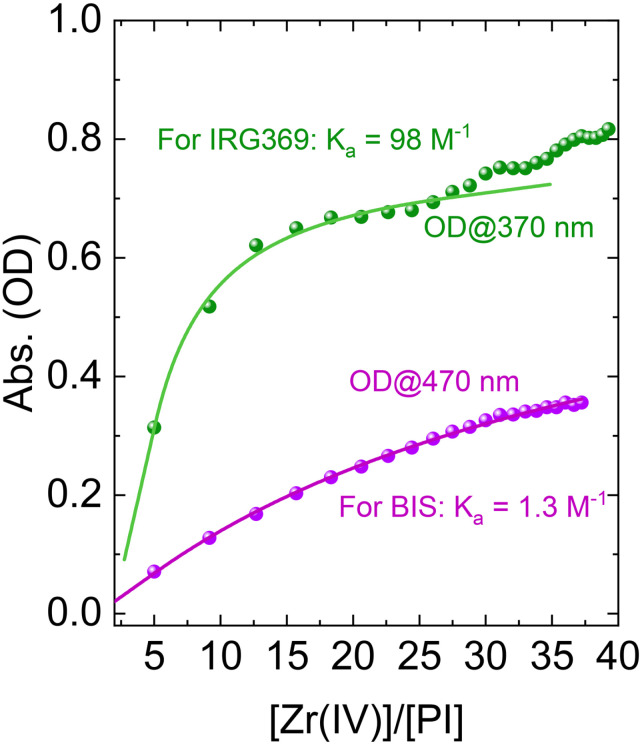
The experimental isotherms (dots) demonstrating the binding between SZ2080™ and IRG369 (green) and BIS (violet). The solid lines represent the best-fits obtained as discussed in ref. 39. The *K*_a_ values denote the association constants of the complexes in both cases. The absorbance was monitored at the maxima of the absorption band of the new species.

The measured binding isotherms to estimate IRG369 and BIS complexation with SZ2080™ are shown in Fig. 6. The overall absorption spectra, used to plot the binding curves are shown in Fig. S7 of the SI. In both cases, the incremental addition of SZ2080™ + PI/MeCN stock, first results in a rapid increase of the product absorption (evolution of the absorption spectra is presented in SI), which eventually slows down, after the concentration of Zr(iv) becomes high enough. The eye-catching feature looking at both isothrems is that in the case of IRG369, this increase is much faster than in the case of BIS, pointing to much stronger binding of IRG369 with SZ2080™ than BIS. To estimate the association constants, we assumed 1 : 1 complexation.^39^ The full fitting procedure and the equation describing a 1 : 1 adduct can be found in ref. 39. Fitting of the isotherms yields *K*_a_ values of 98 M^−1^ and 1.3 M^−1^ for the association of SZ2080™ with IRG369 and BIS, respectively. For IRG369 complexes, only the titration points below 25 equivalents have been considered, as higher concentrations of Zr(iv) may lead to the formation of higher-order adducts, evident from the change in curvature of the experimental points. The observed binding constant for IRG369 is in line with NMR observations, pointing to the formation of a stronger complex by both the carbonyl and NMe_2_ groups. Note that titration experiments were carried out in acetonitrile, thus suggesting that these complexes are stable in this particular solvent.

Further support of the hypothesis that the PIs and SZ2080™ form a donor–acceptor for IRG369 and BIS can be easily achieved by introducing a competing ligand, such as triethylamine (TEA), which in turn should disrupt the complex. Both IRG369 and BIS are less electron-rich than TEA, and therefore TEA donates an electron pair from its nitrogen atom to the Zr(iv) atom in the SZ2080™ framework. This share of electrons exchanges the binding between PI and the Zr atom, ultimately leading to the breakdown of the PI–SZ2080™ complex and the recovery of the (unmodified) ground-state absorption. The ground-state absorption spectra of MeCN solutions with and without TEA are presented in Fig. S8 of the SI. As expected, upon addition of TEA in a 1 : 1 ratio to the volume of MeCN in the solution, we recover the unmodified spectrum resulting from the addition of both component spectra. This strongly suggests that the observed spectral changes are associated only with Zr(iv) in the inorganic network of SZ2080™ and, along with NMR observations, allow us to draw a potential structure of the complexes obtained in both cases (see the insets of Fig. 5A and B). Additionally, we observed that the absorption and fluorescence spectra are slightly altered by the solvent, indicating that these complexes have a donor–acceptor origin (see Fig. S9 and S10 of the SI).

The nearly two-order-of-magnitude difference in the association constants (*K*_a_) between IRG369 (98 M^−1^) and BIS (1.3 M^−1^) can be directly attributed to the thermodynamic chelate effect. As supported by the NMR titrations (Section 3.4), the molecular geometry of IRG369 places its carbonyl and dimethylamino groups in close proximity, allowing it to simultaneously coordinate the Zr(iv) center at two sites. This bidentate binding forms a stable coordination ring, which is entropically highly favorable compared to the binding of a single continuous ligand. In contrast, the spatial separation of the electron-donating groups in BIS sterically restricts it to binding strictly as a weaker, monodentate ligand. This fundamental difference in coordination geometry perfectly explains the greater thermodynamic stability (reflected in the higher binding affinity) of the IRG369 complex.

### 3.6 Density functional theory calculations

**Fig. 7:**
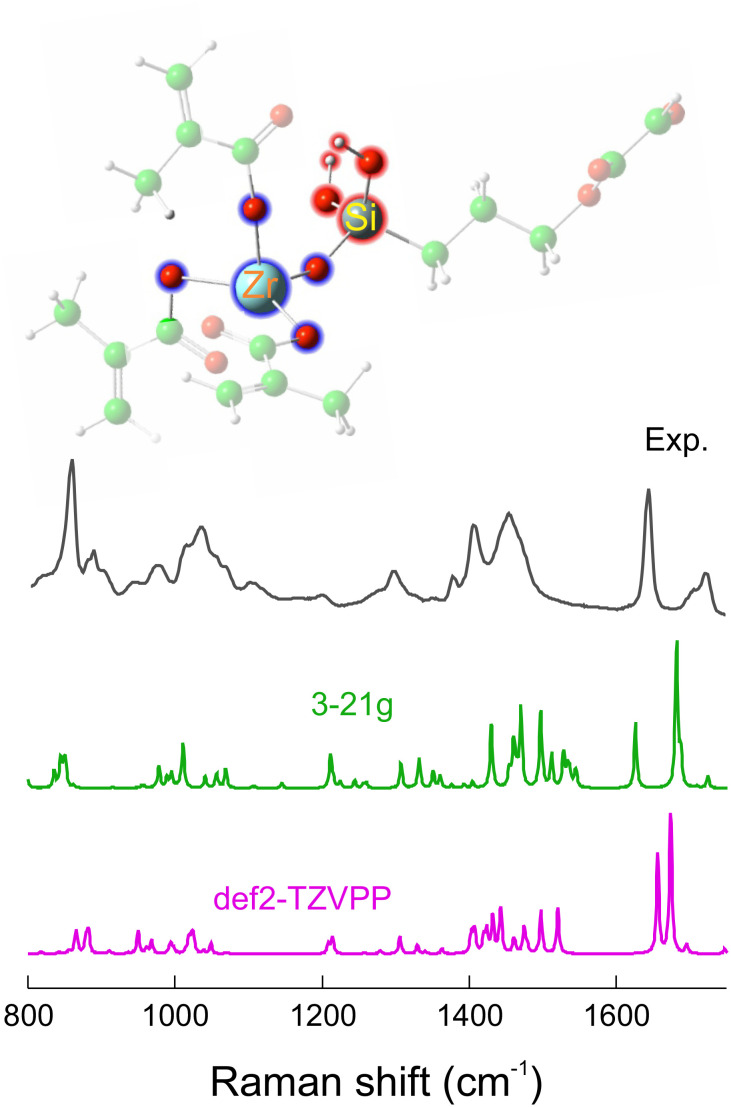
Experimental (black) and calculated Raman spectra of SZ2080™ using different basis sets. The inset shows the optimised structure of SZ2080™ relevant for this study. The calculated frequencies as well as the Raman activities were scaled as described in ref. 40.

Density functional theory calculations (DFT) were used to identify the Raman spectral bands and associate them with the vibrations of SZ2080™ prepolymer. The calculated Raman spectrum with the optimised SZ2080™ structure is presented in Fig. 7. It is worth noting that the calculated spectrum exhibits a larger number of vibrational lines than observed experimentally, likely due to the limited experimental spectral resolution. Nevertheless, the simulated and experimental spectra show reasonable agreement at several characteristic vibrational modes. The main relevant modes are listed in the SI (Table S1).

### 3.7 Implications for photopolymerisation

The observation of MPP indicates that microfabrication conditions are influenced by the PI ability to form a ground-state complex. When such a complex is formed, photoinitiation should become diffusion-independent, enabling more localised photopolymerisation. In contrast, BAPO and TPO show no coordination with SZ2080™ (see Fig. S11 for supplementary Raman data), so as we expect from the photochemistry of these PIs,^41^ the initiation relies on radical diffusion and produces a significantly narrower DFW. Donor–acceptor complex formation between hybrid prepolymers and electron-rich PIs introduces an alternative initiation mechanism that can accelerate polymerisation so increasing the writing speed as well as enhance DFW. In hybrid organic–inorganic prepolymers containing Zr(iv), electron-donating PIs can coordinate with the inorganic network, localising reactive species and enabling immediate, diffusion-independent polymer chain growth. Prior work has shown that Zr-based hybrid resins work at high writing speeds,^42^ which may result from reduced diffusion during initiation. Overall, the development of these ground-state complexes by coupling transition-metal-containing hybrid resins with electron-rich PIs offers a promising strategy to improve control in MPP. The easiest way to monitor the formation of such complexes was UV/Vis absorption spectroscopy, along with emission measurements that clearly pointed out the differences occurring due to the complexation of the photoinitiator and the SZ2080™ prepolymer.

## 4 Conclusions

In conclusion, the results presented in this study have established a clear link between the fabrication window and the formation of ground-state complexes between SZ2080™ prepolymer and electron rich IRG369 and BIS photoinitiators. The totality of spectroscopic structural data points towards the formation of a donor–acceptor complex between the Zr–Si–O chain in the SZ2080™ prepolymer, and the Lewis basic **N**R_2_ or C=**O** atoms of the PI. We suggest that the improvement of polymerisation performance is due to elimination of diffusion-limited reaction steps between the SZ2080™ prepolymer and the PIs. Since the ground state complexes are readily observed by their characteristic changes in the optical spectra, the presence of such changes could be used as a guide in design and testing of new material combinations for MPP. Our study thus opens avenues for further exploration of hybrid organic–inorganic materials in additive manufacturing, highlighting the role and importance of coordination between PI and resin, towards optimising photopolymerisation processes.

## Author contributions

MN and MV conceived the study and developed the methodology (conceptualisation, methodology). MN, MV conducted SRS experiments, including data analysis. DL prepared the samples. EO performed NMR measurements and NMR data analysis. MT and GN conducted the conventional Raman and FTIR measurements and analysed the associated data. MGr and MT performed DFT calculations. MGa conducted laser fabrication. MN, RFT conducted fluorescence measurements and UV/Vis titration experiments. MN managed the data and, together with MV, wrote the manuscript. RFT, MM reviewed and edited the manuscript. MV, MM and RFT acquired funding for the project.

## Conflicts of interest

There are no conflicts to declare.

## Supplementary Material

MA-007-D5MA01526J-s001

## Data Availability

The data that support the findings of this study are available in the supplementary information (SI) of this article. Supplementary information is available. See DOI: https://doi.org/10.1039/d5ma01526j.
