# Supplementary material for: Donor–acceptor complexes between photoinitiators and hybrid organic–inorganic SZ2080™ photoresist
Source: Mater Adv. 2026 Mar 13;7(7):3864–74. doi: 10.1039/d5ma01526j (PMC12998534; doi:10.1039/d5ma01526j)
Supplement: MA-007-D5MA01526J-s001 [file MA-007-D5MA01526J-s001.pdf]

Supplementary Information (SI) for Materials Advances.
This journal is © The Royal Society of Chemistry 2026

Supplementary information

Donor–Acceptor Complexes between Photoinitiators and Hybrid Organic–Inorganic SZ2080™ Photoresist

Marius Navickas,[a] Dimitra Ladika,[a] Edvinas Orentas,[b] Martynas Talaikis,[b] Gediminas Niaura,[b] Mantas Grigalavičius,[a] Mantas Gaidys,[a] Ricadro J. Fernández-Terán,*[c] Mangirdas Malinauskas[a] and Mikas Vengris[a]*

[a] Vilnius University, Laser Research Center, Saulėteko av. 10, LT-10223 Vilnius, Lithuania; E-mail: marius.navickas@ff.vu.lt

[b] Center for Physical Sciences and Technology, Saulėtekio av. 3, LT-10257 Vilnius, Lithuania;

[c] Department of Physical Chemistry, University of Geneva, CH-1205 Geneva, Switzerland; E-mail: Ricardo.FernandezTeran@unige.ch

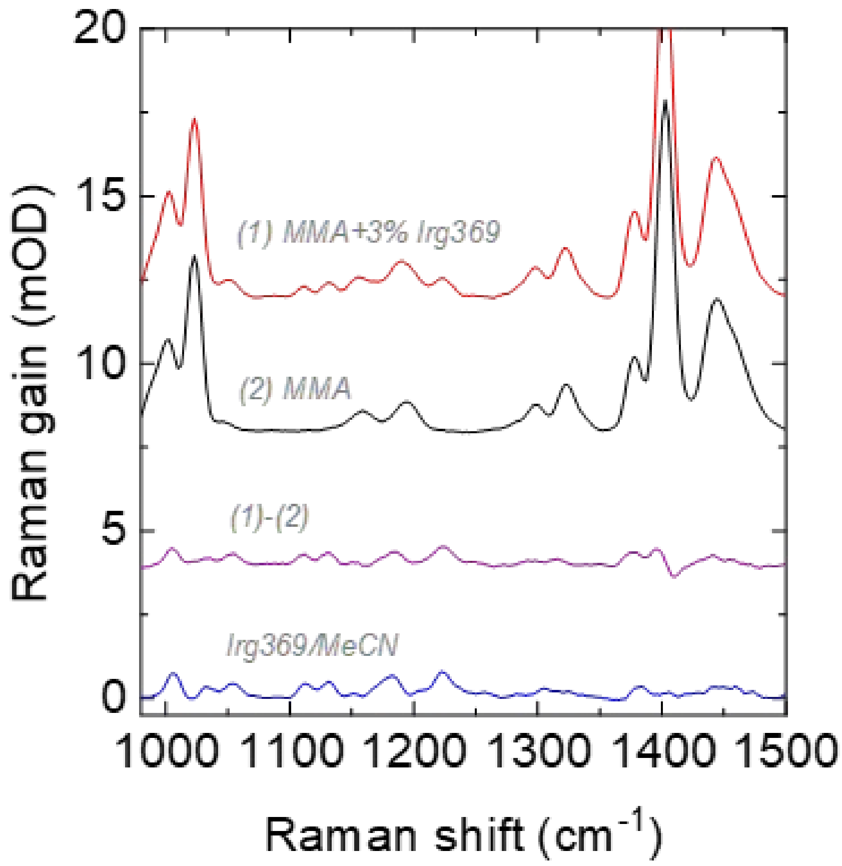

Fig. S1. SRS spectra of MMA, sensitised with IRG369. The black curve represents the SRS spectrum of the pure resin, while the red curve indicates the SRS spectrum of the resins containing 3% photoinitiator. The violet and blue curves show the difference in SRS spectra between the mixture and pure resin, as well as the SRS spectra of the pure PI dissolved in MeCN. The Raman pump wavelength was 800 nm.

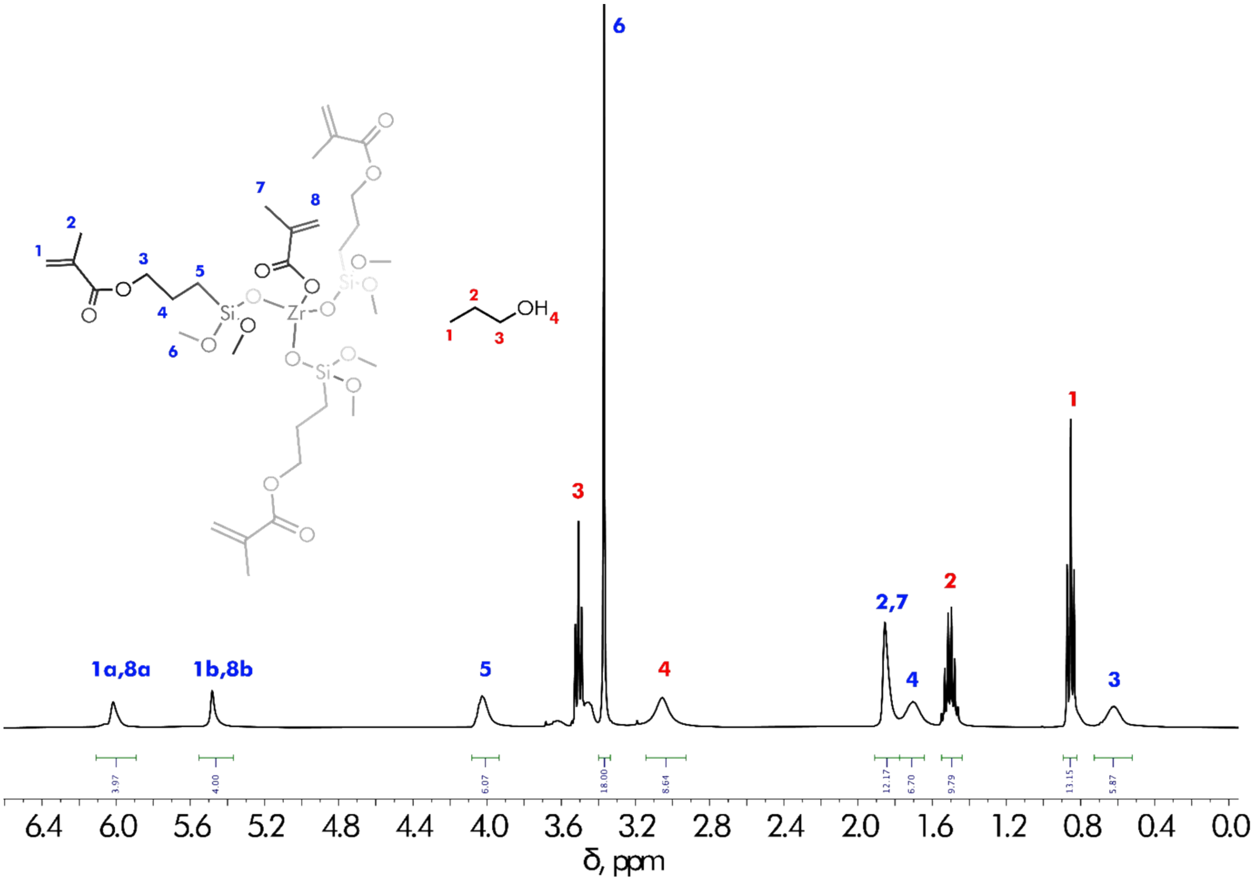

Fig. S2. ^{1}H NMR ($CDCl_3$ 400 MHz) spectrum of SZ2080™ Double bond protons 1,8 and methyl group protons 2,7 have identical chemical shifts. Sharp and well-resolved signals belong for propanol molecules.

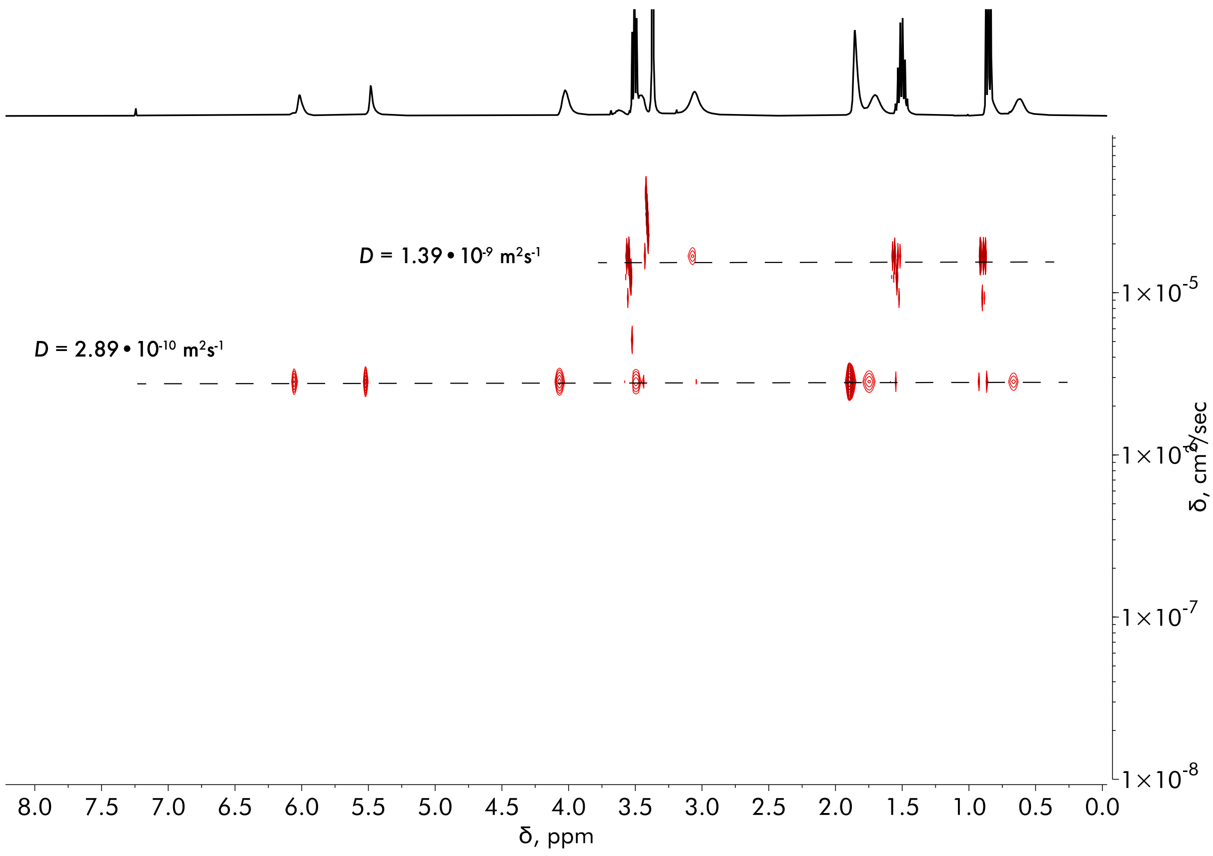

Fig. S3. DOSY ($CDCl_3$, 400 MHz) spectrum of SZ2080™. A higher value of the diffusion coefficient for the propanol signals confirms that it is not bound to zirconium metal.

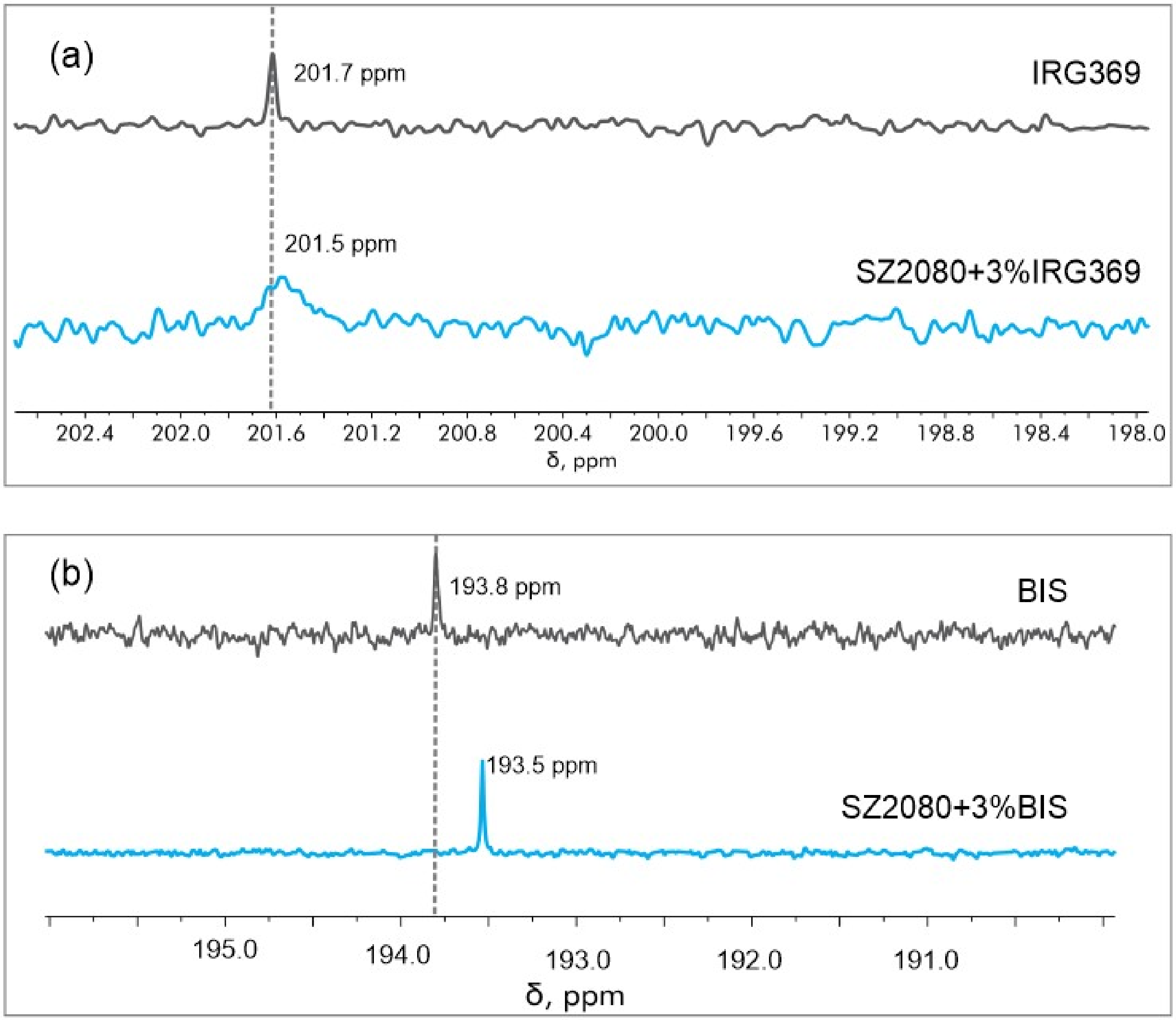

Fig. S4. ^{13}C(*C=O) NMR spectra (CD_3CN, 101 MHz) of (a) IRG369 and SZ2080^{TM} + 3 wt% IRG369 and (b) IRG369 and SZ2080^{TM} + 3 wt% BIS.

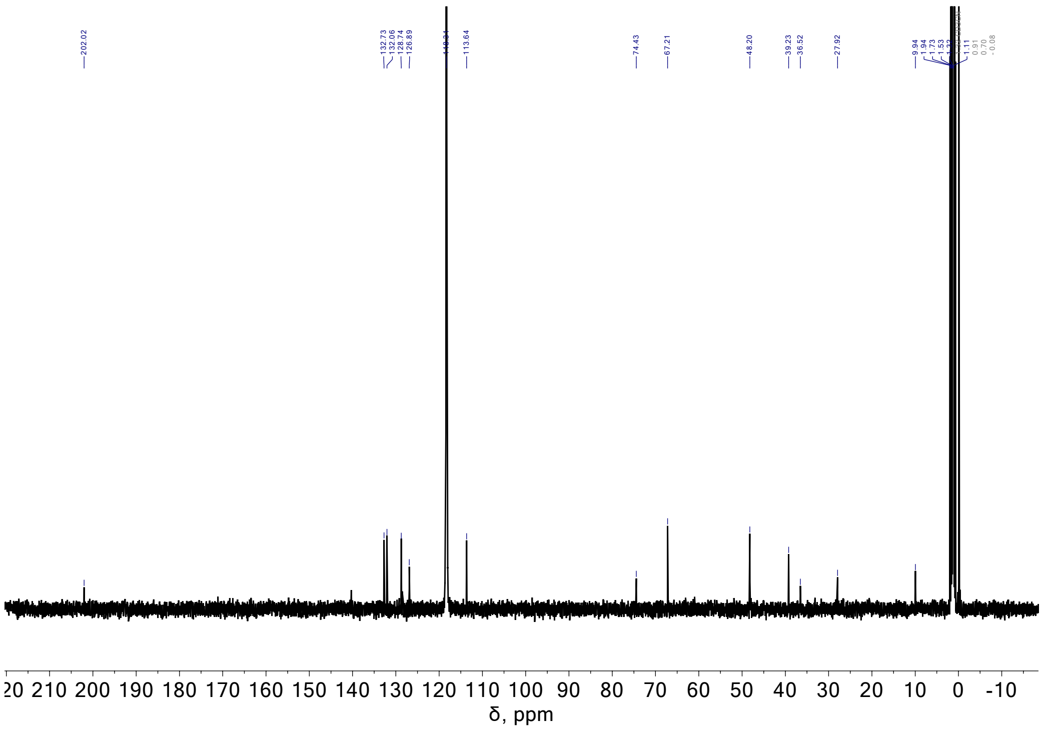

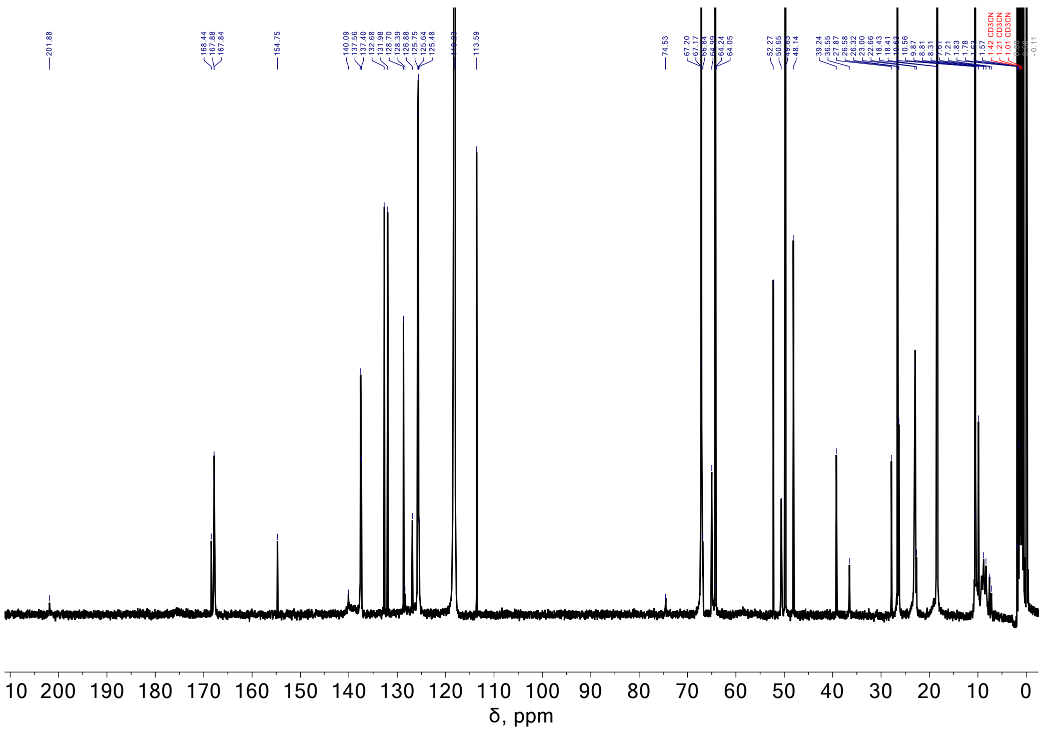

Fig. S5. ^{13}C NMR (CD_3CN, 101 MHz) of IRG369 (top) and SZ2080™ + 3 wt% IRG369 (bottom).

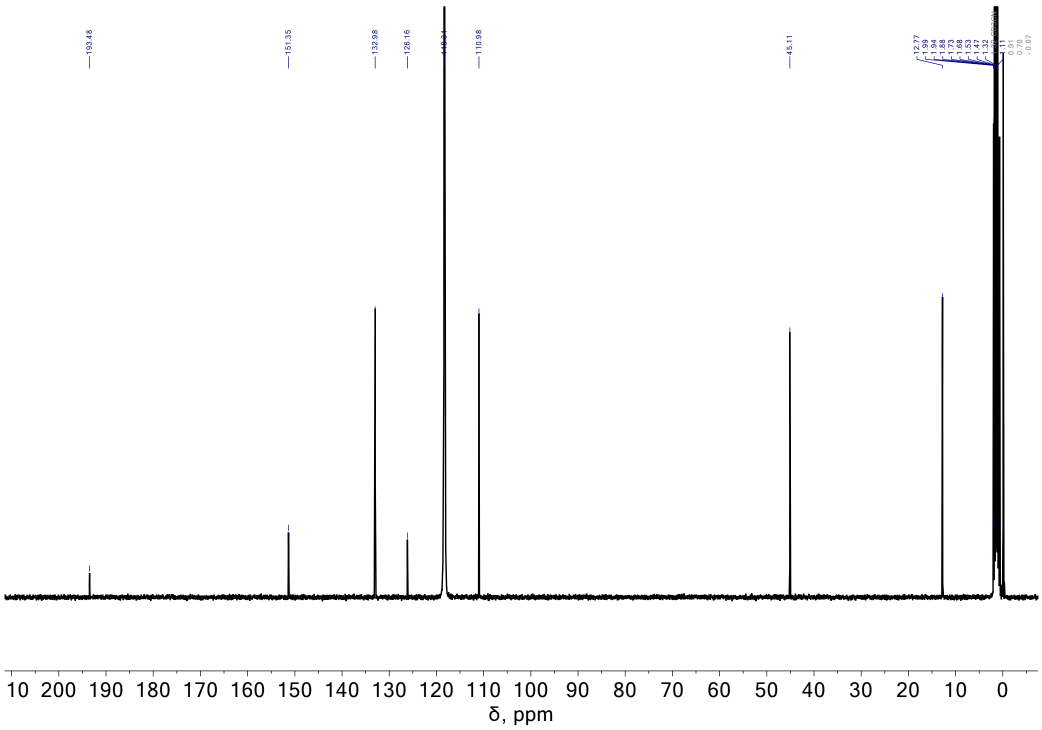

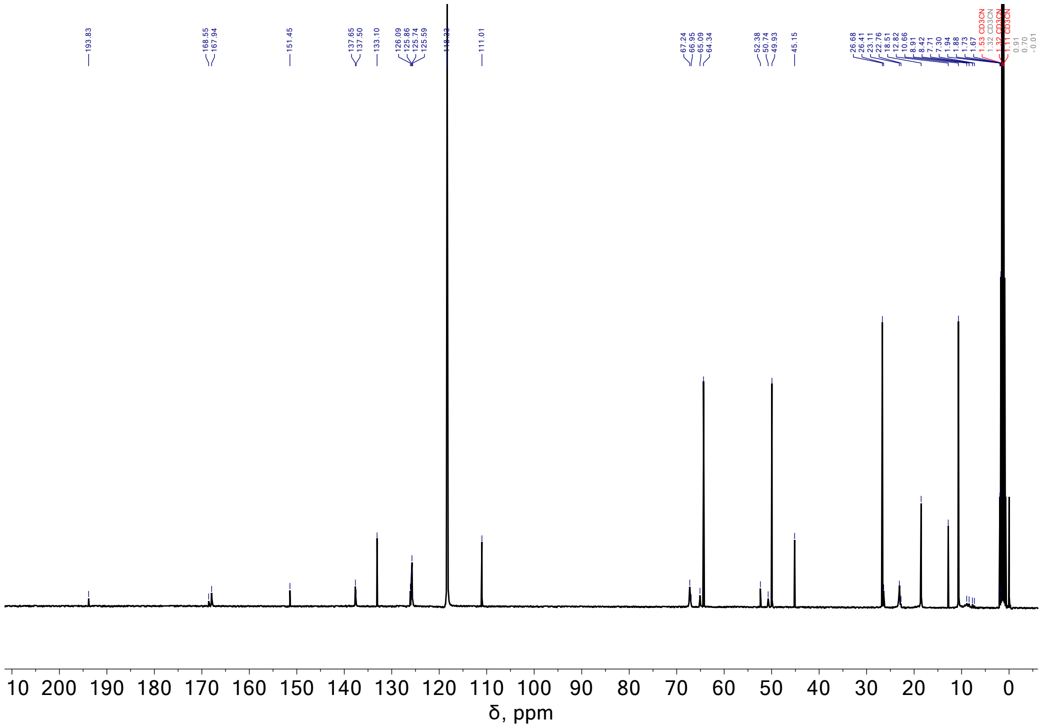

Fig. S6. ^{13}C NMR (CD_3CN, 101 MHz) of BIS (top) and SZ2080™ + 3 wt% BIS (bottom).

Table S1. Assignment of the Raman frequencies by DFT calculations of SZ2080™.

Raman (cm^{-1})	DFT (cm^{-1})	Assignment
380	345	δ(CCC), δ(COO), ν(Zr–O)
605	605	δ(COO), ν(Zr–O)
860	865, 881	δ(COO), δ(CCC), ν(Zr–O)
1035	1023	r(CH_3), ν(C–C)
1298	1306	ν(C–C), ν(C–O)
1406	1406	δ_s(CH_3), ν(C–CH_3)
1454	1442	δ(CH_2), δ(CH_3)
1650	1656, 1674	ν(C=C)
1736	1794	ν(C=O)

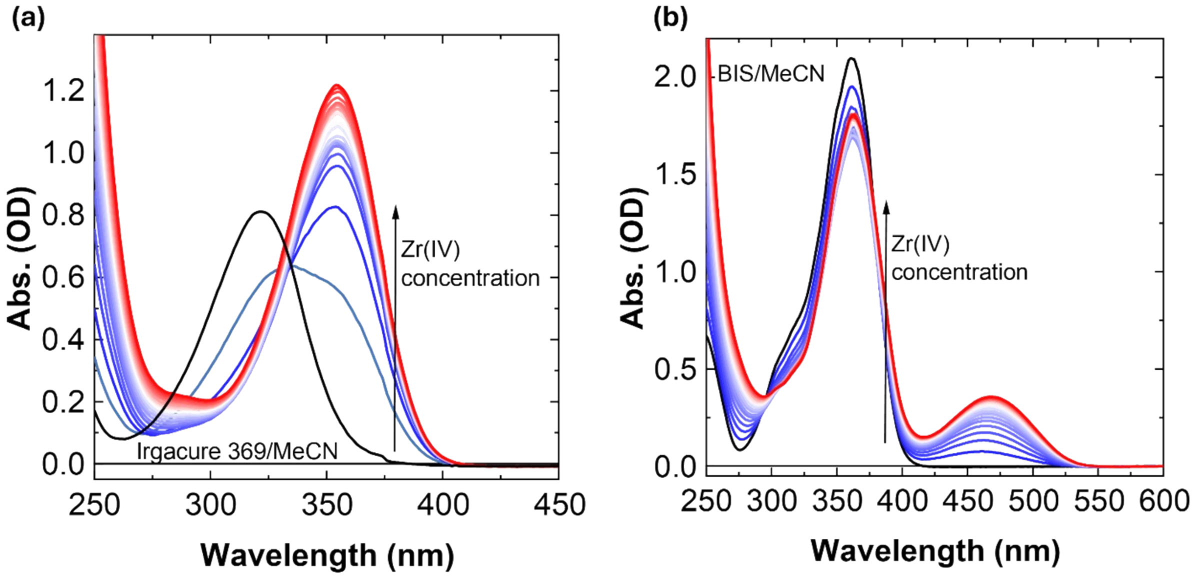

Fig. S7 Absorption changes of (a) IRG369 (Irgacure 369) and (b) BIS photoinitiators upon titration with SZ2080™.

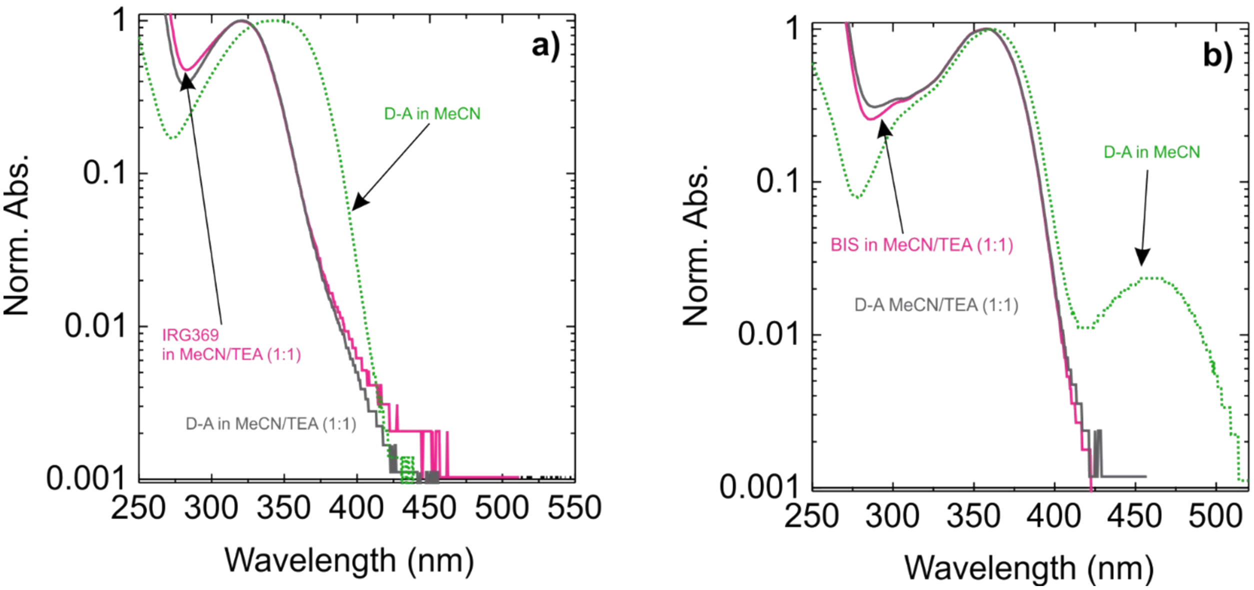

Fig. S8 Ground-state absorption spectra of (a) IRG369-SZ2080™ and (b) BIS-SZ2080™ complexes (D-A), along with the absorption spectra of the corresponding pure PIs in MeCN and MeCN/TEA solutions. All TEA mixtures were prepared with a 1:1 ratio of MeCN to TEA. TEA denotes triethylamine.

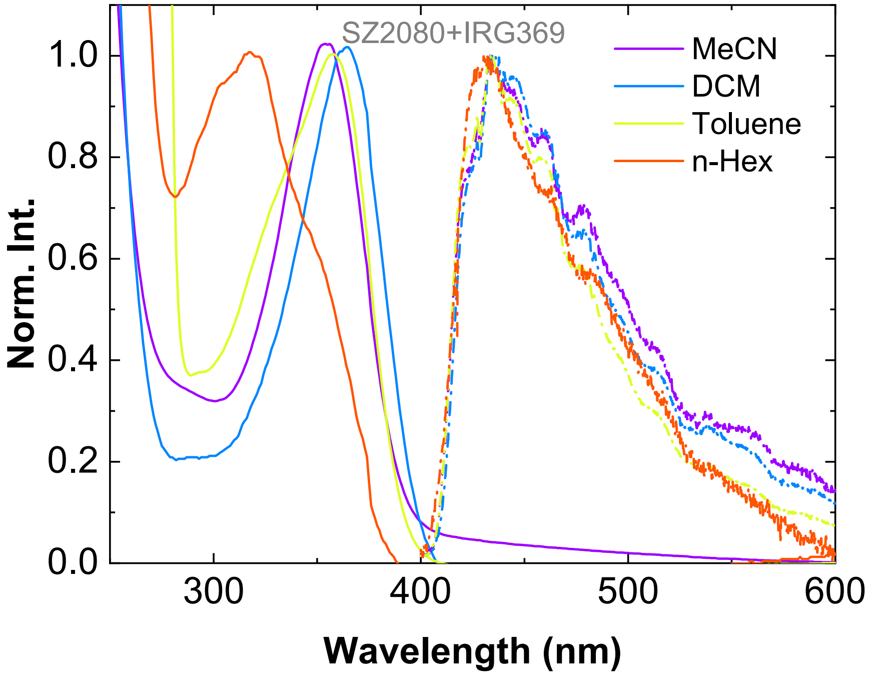

Fig. S9. Ground-state absorption and fluorescence spectra of IRG369-SZ2080™ complexes obtained in different solvents.

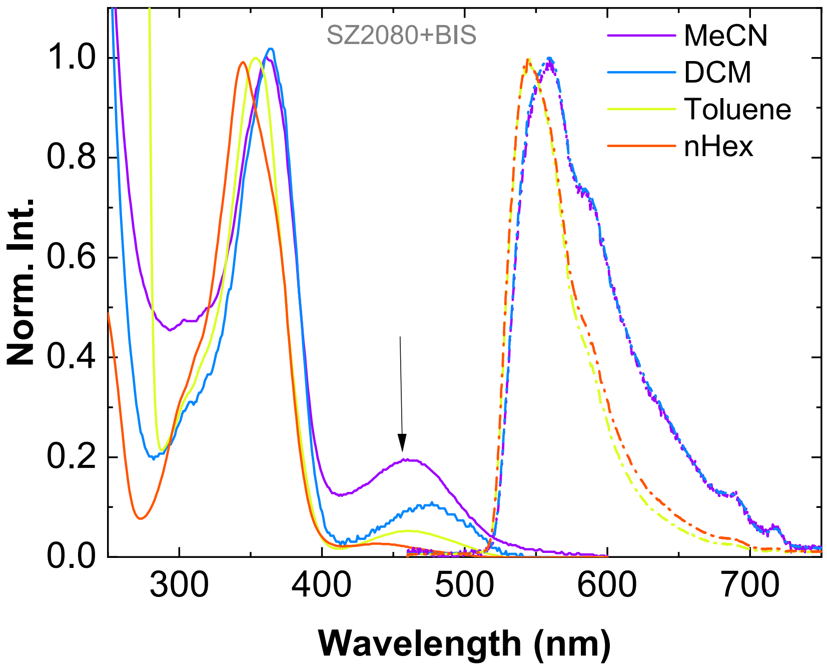

Fig. S10. Ground-state absorption and emission spectra of BIS-SZ2080™ complexes obtained in different solvents.

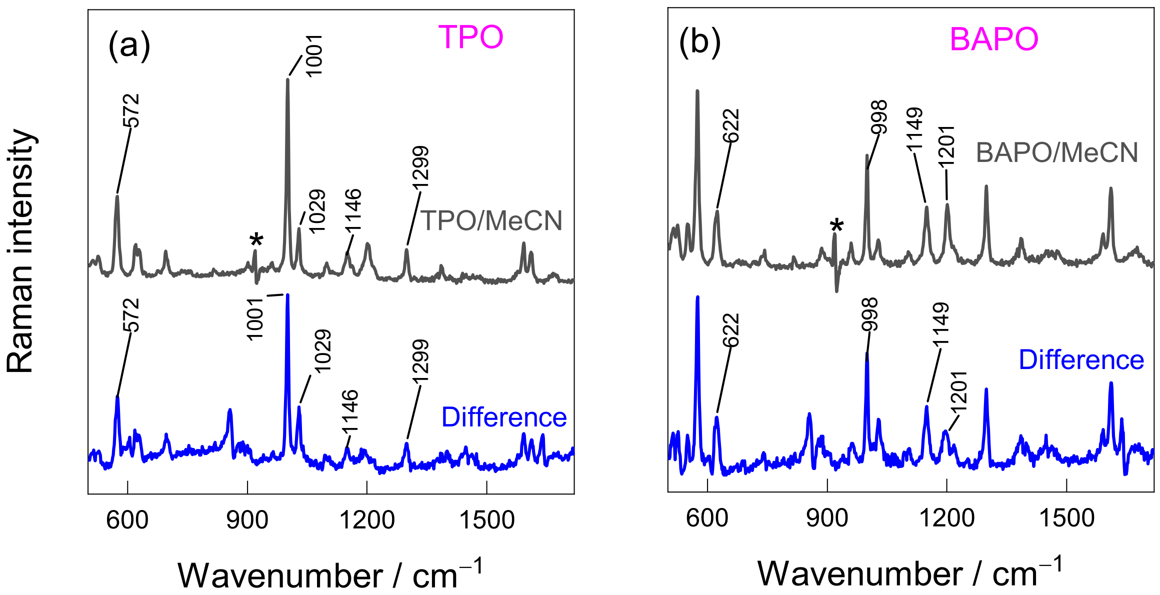

Fig. S11 Comparison of Raman spectra (λ_{ex} = 830 nm) of SZ2080™ sensitised with 3% of (a) TPO and (b) BAPO photoinitiators. The blue curve shows the difference spectrum between sensitised and unsensitised SZ2080™, while the grey curve represents the Raman spectrum of the photoinitiator in MeCN. The asterisks indicate the artefacts due to the subtraction of the solvent.
